# Effect of Antibiotic Therapy Before Arthrocentesis on Synovial Fluid Cell Count and Differential for Diagnosis of Native Joint Septic Arthritis

**DOI:** 10.1093/ofid/ofae403

**Published:** 2024-07-19

**Authors:** Ryan B Khodadadi, Pansachee Damronglerd, Jack W McHugh, Said El Zein, Brian D Lahr, Brandon J Yuan, Omar M Abu Saleh, Gina A Suh, Aaron J Tande

**Affiliations:** Division of Public Health, Infectious Diseases, and Occupational Medicine, Mayo Clinic, Rochester, Minnesota, USA; Division of Public Health, Infectious Diseases, and Occupational Medicine, Mayo Clinic, Rochester, Minnesota, USA; Division of Infectious Diseases, Department of Internal Medicine, Thammasat University, Pathum Thani, Thailand; Division of Public Health, Infectious Diseases, and Occupational Medicine, Mayo Clinic, Rochester, Minnesota, USA; Division of Public Health, Infectious Diseases, and Occupational Medicine, Mayo Clinic, Rochester, Minnesota, USA; Department of Biomedical Statistics and Informatics, Mayo Clinic, Rochester, Minnesota, USA; Department of Orthopedic Surgery, Mayo Clinic, Rochester, Minnesota, USA; Division of Public Health, Infectious Diseases, and Occupational Medicine, Mayo Clinic, Rochester, Minnesota, USA; Division of Public Health, Infectious Diseases, and Occupational Medicine, Mayo Clinic, Rochester, Minnesota, USA; Division of Public Health, Infectious Diseases, and Occupational Medicine, Mayo Clinic, Rochester, Minnesota, USA

**Keywords:** antibiotics, arthrocentesis, cell count and differential, native joint septic arthritis

## Abstract

We examined the effect of preoperative antibiotic exposure and duration on synovial fluid samples from patients with native joint septic arthritis of the hip/knee. While exposure before diagnostic arthrocentesis did not affect fluid parameters, increased duration was associated with a decreased total nucleated cell count, underscoring the complex antibiotic effects on synovial fluid parameters.

## Graphical Abstract

**Graphical Abstract:**
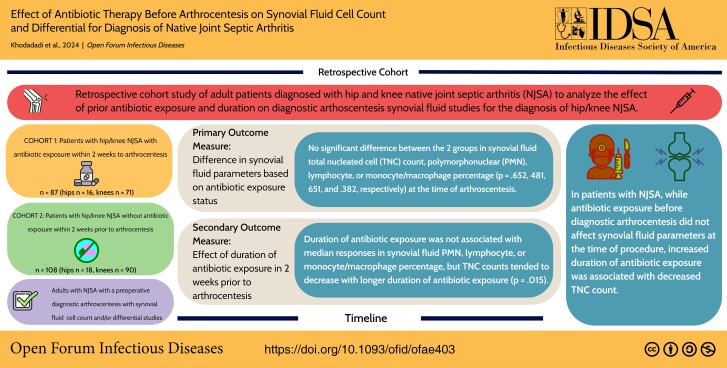
This graphical abstract is also available at Tidbit: https://tidbitapp.io/tidbits/effect-of-antibiotic-therapy-before-arthrocentesis-on-synovial-fluid-cell-count-and-differential-for-diagnosis-of-native-joint-septic-arthritis-3c2a7a4f-75e8-487a-94fc-c2a432c7adf9

Native joint septic arthritis (NJSA), most often affecting large joints such as hips and knees, is a medical condition associated with significant morbidity and mortality if not promptly diagnosed and treated [1–4]. Timely identification of a causative organism is crucial, and diagnostic arthrocentesis plays a pivotal role by allowing for direct synovial fluid analysis of the affected joint with testing such as cell count with differential, Gram stain, and culture prior to surgical drainage [5].

However, antibiotic administration can limit the diagnostic accuracy of arthrocentesis. Recent reports have demonstrated that antibiotics administered before joint aspiration for suspected NJSA reduce the synovial white blood cell count, polymorphonuclear neutrophil (PMN) percentage, and rate of culture positivity, adding to the literature surrounding the effect of antibiotics on arthrocentesis for diagnosis of NJSA [6, 7].

While the binary impact of antibiotic therapy has been evaluated in other studies, the effect of antibiotic exposure and duration on large-joint synovial fluid parameters is less well studied. Given this gap in the literature, we sought to elucidate the effect of prior antibiotic exposure and duration on arthrocentesis synovial fluid studies for the diagnosis of large-joint NJSA.

## METHODS

### Study Design

Following institution internal review board approval (22-005205), we performed a retrospective cohort study of adult patients diagnosed with hip and knee NJSA who underwent preoperative arthrocentesis at Mayo Clinic Hospitals in Arizona, Florida, Minnesota, and Wisconsin between January 2012 and December 2021. These patients originated from a larger cohort of NJSA cases (557 joints from 496 patients) described in a previous study [8]. The exclusion criteria for that study included a lack of research authorization per Minnesota statute, limited medical records, loss to follow-up, an alternative diagnosis, prosthetic joint infection, infection of an excluded joint location, or prior placement of hardware/excision of the affected joint. For the current study, the cohort was limited to 195 cases of hip and knee NJSA that had at least 1 of the following 4 synovial fluid parameters measured: total nucleated cell (TNC) count and PMN, lymphocyte, and monocyte/macrophage percentage.

Following identification of eligible patients, data were manually extracted from the electronic medical record to a prespecified form via REDCap as previously described [8]. Abstracted data included patient demographics, comorbidities, clinical features, and synovial fluid studies from diagnostic arthrocentesis at the time of presentation to our facility.

### Definitions

Cases of NJSA were defined by meeting 1 of the modified Newman criteria: (1) isolation of a pathogenic organism from an affected joint, (2) isolation of a pathogenic organism from another source (blood) in the context of a hot red joint suspicious for sepsis, (3) typical clinical features and turbid joint fluid in the presence of prior antibiotic treatment, or (4) postmortem or pathologic features suspicious for NJSA [9]. Patients with hip or knee NJSA were divided into 2 groups based on antibiotic exposure status (within 2 weeks) as assessed through comprehensive medical administration records prior to arthrocentesis. Duration of antibiotic exposure was quantified in days of completed therapy based on the dosing schedule of the antibiotic administered, adjusted for creatinine clearance. Synovial fluid TNC count and PMN, lymphocyte, and monocyte/macrophage percentage were compared between groups to assess the effect of antibiotic therapy on synovial fluid parameters. We also analyzed the effect of antibiotic duration, up to 2 weeks prior to diagnosis, on median synovial fluid parameters.

### Statistical Analysis

Descriptive statistics were presented as median with IQR for continuous variables and frequency with percentage for categorical variables. Wilcoxon rank sum tests were used to compare the synovial fluid parameters in the groups with and without prior antibiotic treatment. In secondary analyses, separate univariable quantile regression models were fitted for these parameters to assess the effect of antibiotic exposure as a continuous measure of duration, ranging from 0 days (ie, no prior antibiotics) to 14 days. Specifically, duration was entered into the regression as a 4-knot restricted cubic spline with a discontinuity at 0 days to allow nonlinear relationships and a single discontinuous vertical shift at zero. All analyses were done with R version 4.1.2 (R Foundation).

## RESULTS

In total, 195 patients diagnosed with NJSA (161 knees and 34 hips) met the study criteria. An overall 108 patients received no antibiotics, whereas 87 were exposed to antibiotics within 2 weeks of arthrocentesis. The median age was 61.9 years (IQR, 53.7–70.4), and most patients were male (n = 128, 65.6%) and White (n = 176, 91.7%). Additional baseline characteristics, comorbidities, and synovial fluid studies are presented in Table 1.

**Table 1.** Characteristics of 195 Patients With Hip and Knee Native Joint Septic Arthritis

		Antibiotics Before Arthrocentesis		
Characteristic	Overall (N = 195)	No (n = 108)	Yes (n = 87)	p-value
Age at time of surgery, years	61.9 (53.5–70.4)	61.6 (52.5–70.4)	63.3 (55.0–70.5)	
Male gender	128 (65.6)	69 (63.9)	59 (67.8)	
Race (n = 192)				
American Indian/Alaska Native	3 (1.6)	1 (0.9)	2 (2.3)	
Asian	6 (3.1)	4 (3.8)	2 (2.3)	
Black or African American	7 (3.6)	4 (3.8)	3 (3.5)	
White	176 (91.7)	97 (91.5)	79 (91.9)	
Body mass index, kg/m²	29.7 (25.1–33.9)	29.0 (25.1–33.8)	30.1 (25.6–33.9)	
CCI with age adjustment	4 (2–6)	4 (2–6)	4 (1–6)	
Joint type				
Hip	34	18 (16.7)	16 (18.4)	
Knee	161	90 (82.6)	71 (81.6)	
Current smoker	27 (13.8)	15 (13.9)	12 (13.8)	
Alcohol dependence	33 (16.9)	18 (16.7)	15 (17.2)	
Current intravenous drug use	4 (2.1)	2 (1.9)	2 (2.3)	
Osteoarthritis of affected joint	76 (39.0)	47 (43.5)	29 (33.3)	
Diabetes	61 (31.3)	31 (28.7)	30 (34.5)	
Gout	21 (10.8)	6 (5.6)	15 (17.2)	
CPPD disease	6 (3.1)	5 (4.6)	1 (1.1)	
Inflammatory arthropathy	17 (8.7)	9 (8.3)	5 (5.7)	
Chronic kidney disease	35 (17.9)	15 (13.9)	20 (23.0)	
End-stage renal disease/dialysis	10 (5.1)	5 (4.6)	5 (5.7)	
Liver disease	20 (10.3)	12 (11.1)	8 (9.2)	
History of Nondermatologic malignancy	28 (14.4)	15 (13.9)	13 (14.9)	
History of Transplant	7 (3.6)	4 (3.7)	3 (3.4)	
Other immunocompromising condition	11 (5.6)	6 (5.6)	5 (5.7)	
Predisposing dermatologic condition	18 (9.2)	6 (5.6)	12 (13.8)	
Steroid use > 20 mg of > 4 weeks	6 (3.1)	4 (3.7)	2 (2.3)	
Duration of symptoms at presentation, days	4 (2–10)	4 (2–10)	5 (2–11)	
Trauma to affected joint	35 (17.9)	16 (14.8)	19 (21.8)	
Skin and soft tissue infection of affected area	18 (9.2)	3 (2.8)	15 (17.2)	
Surgery to affected joint				
Recent: < 3 months	12 (6.2)	6 (5.6)	6 (6.9)	
Prior: > 3 months	13 (6.7)	9 (8.3)	4 (4.6)	
Steroid injection to affected joint				
Recent: < 3 months	24 (12.3)	16 (14.8)	8 (9.2)	
Prior History: > 3 months	6 (3.1)	5 (4.6)	1 (1.1)	
Duration of antibiotics prior to aspiration, days	0.0 (0.0–1.4)	0.0 (0.0–0.0)	2.0 (0.5–4.5)	
Synovial Fluid Parameters				
Total Nucleated Cell Count, /mcL (n = 194)	63,824 (36 270–105 491)	62,988 (38 326–96 622)	64,932 (35 756–121 144)	.652
≥50,000 /mcL	122 (62.9)	70 (64.8)	52 (60.5)	.533
PMNs, % (n = 194)	92.0 (86.0–95.0)	91.0 (87.5–94.5)	92.0 (84.5–95.5)	.481
Lymphocytes, % (n = 163)	2.0 (1.0–5.0)	2.5 (1.0–5.0)	2.0 (1.0–5.0)	.651
Monocytes/macrophages, % (n = 174)	6.0 (3.0–10.0)	6.0 (3.0–10.0)	5.0 (3.0–10.5)	.382

Data are presented as No. (%) or median (IQR).

Abbreviations: CCI, Charlson Comorbidity Index; CPPD, calcium pyrophosphate deposition; PMN, polymorphonuclear neutrophil.

Synovial fluid parameters are also shown in Table 1, overall and by group. Among patients exposed to antibiotics, the median duration of exposure before arthrocentesis was 2.0 days (IQR, 0.5–4.5). There was no significant difference between the groups in synovial fluid TNC count or PMN, lymphocyte, or monocyte/macrophage percentage (*P* = .652, 481, 651, and .382, respectively) at the time of aspiration.

**Figure 1.**
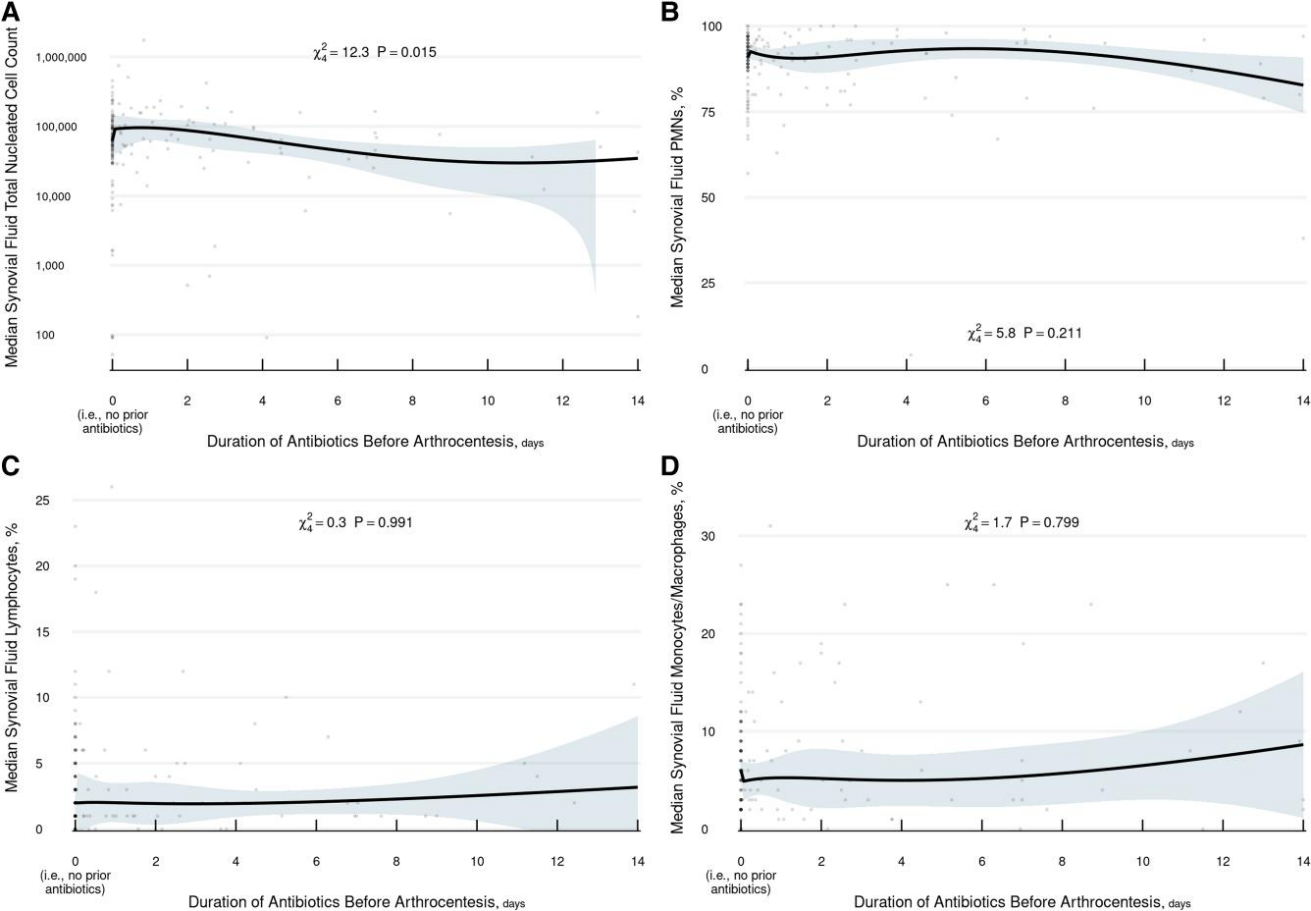
Effect of antibiotic duration on synovial fluid parameters: *A*, total nucleated cell count; *B*, polymorphonuclear neutrophils; *C*, lymphocytes; *D*, monocytes/macrophages.

We performed secondary analyses to elucidate the effect of duration of antibiotic exposure in the 2 weeks prior to arthrocentesis in patients with or without documented antibiotic exposure. Antibiotic duration was not associated with median responses in synovial fluid PMN, lymphocyte, or monocyte/macrophage percentage, but it was associated with TNC count (*P* = .015). Specifically, predicted median TNC counts tended to decrease with longer duration of antibiotic exposure (Figure 1).

## DISCUSSION

Our study evaluated the impact of prior antibiotic exposure and duration within 2 weeks on synovial fluid studies in patients with confirmed hip and knee NJSA. Interestingly, we did not observe a statistically significant difference in synovial fluid median TNC count or PMN, lymphocyte, and monocyte/macrophage percentage among patients based on antibiotic exposure status at the time of arthrocentesis, as noted in recent reports [6, 7]. However, secondary analysis revealed that longer courses of antibiotics before arthrocentesis were associated with decreasing median TNC counts. In addition, there does not appear to be a decline in median PMN percentage with increasing duration of antibiotic exposure, suggesting that sustained derangement of this parameter may remain a valuable clinical marker of NJSA in this clinical context.

Prior studies comparing patients grouped by antibiotic exposure status with a similar percentage of patients receiving antibiotics before arthrocentesis demonstrated an antibiotic effect on Gram stain positivity, culture yield, synovial fluid white blood cell count, and PMN percentage but did not exclusively assess NJSA or distinguish between large and small NJSA [6, 7, 10]. However, these factors require careful assessment, given the difference in expected cell counts based on the presence of joint arthroplasty or joint size [11–15]. For these reasons, we chose to focus on a single syndrome (NJSA) in a uniform joint population (large joints).

In addition, prior studies have approached antibiotic therapy as a binary exposure variable, which does not reflect the full impact of therapy on cell count and differential over time [6, 7]. Furthermore, patients in these reports received fewer antibiotic doses, and data surrounding antibiotic exposure were in some cases limited to the 24-hour time frame prior to diagnostic arthrocentesis [6, 7].

Following review of robust historical clinical and medical administration records data, we were able to assess antibiotic exposure over a 2-week interval before presentation to elucidate the impact of antibiotic exposure duration prior to diagnostic arthrocentesis. Our findings—specifically, the effects of antibiotic exposure and duration on synovial fluid parameters—contribute to the existing literature by highlighting that, with increasing duration of antibiotic exposure, there appears to be a decrease in median TNC count but no relation with median PMN differential derangement in hip and knee NJSA, highlighting the complex influence of preceding antibiotics on the synovial fluid parameters. These findings underscore the need to individualize the interpretation of these parameters in clinical practice by taking into consideration the duration of antibiotic exposure at the time of clinical presentation.

Potential implications of these findings on clinical care include maintaining a high index of clinical suspicion for NJSA for patients exposed to antibiotics when reviewing synovial fluid studies: although prolonged antibiotic exposure may decrease the TNC count, alterations in differential, including PMN percentage, may be persistent and suggestive of infection. Taking these findings into account, as well as other studies outlining the effect of antibiotics on the diagnosis of NJSA, we propose obtaining peripheral blood cultures and arthrocentesis, if possible, before initiating antibiotic therapy at the time of medical attention, aligning with our previously outlined approach in a study describing the effect of preoperative antibiotic therapy on subsequent operative culture yield for the diagnosis of NJSA [8].

The strengths of our study include a relatively large cohort of NJSA of similar joints with detailed assessment of the effect of antibiotics on synovial fluid studies through analysis of antibiotic exposure and duration in inpatient and outpatient clinical settings. Limitations include the retrospective nature of our study, introducing the potential for bias and confounding not accounted for in analyses. Furthermore, the use of a 2-week cutoff for antibiotic exposure may not capture the full spectrum of the effect of antibiotic therapy on synovial fluid parameters.

In conclusion, our study adds to the growing body of literature on the impact of antibiotic therapy on arthrocentesis for diagnosis of NJSA. While our primary analysis suggests that synovial fluid parameters remain similar with and without the influence of preceding antibiotics, our secondary analysis revealed a significant association for the duration of antibiotic exposure with TNC count but not PMN percentage. These findings highlight the need for further research to better understand the complex relationship between antibiotic therapy and synovial fluid parameters in the diagnosis of NJSA, with future efforts dedicated to clarifying mixed findings in the literature or offering novel diagnostic studies to aide in the diagnosis of this condition.

## Notes

***Author contributions.*** R. B. K., B. D. L., and A. J. T. designed the study. R. B. K., P. D., J. W. M., and S. E. Z. performed data collection and processing. B. D. L. analyzed the data. R. B. K., B. D. L., and A. J. T. reviewed data analysis. R. B. K. wrote the first draft of the manuscript. All authors contributed to and reviewed the final manuscript.

***Patient consent statement.*** The study was performed following approval from the Mayo Clinic Institutional Review Board under a waiver of informed consent (22-005205) on 20 June 2022.

***Financial support.*** This work was supported by the National Center for Advancing Translational Sciences (grant UL1 TR002377).
